# Supplementary material for: Brain Tissue-Derived Extracellular Vesicle Mediated Therapy in the Neonatal Ischemic Brain
Source: Int J Mol Sci. 2022 Jan 6;23(2):620. doi: 10.3390/ijms23020620 (PMC8775954; doi:10.3390/ijms23020620)
Supplement: Supplementary file 1 [file ijms-23-00620-s001.zip › ijms-1512598-supplementary.pdf]

Supplemental Material

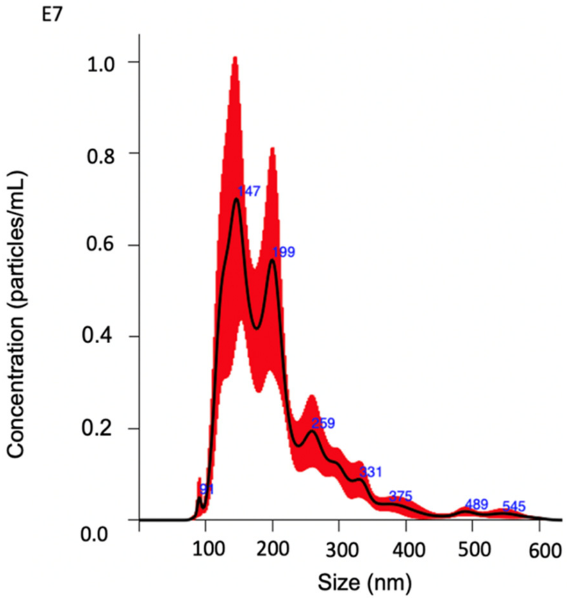

Figure S1. Low magnification TEM images of BEV clusters using a negative staining technique. White arrows indicate BEVs. Scale bar = 100 nm.

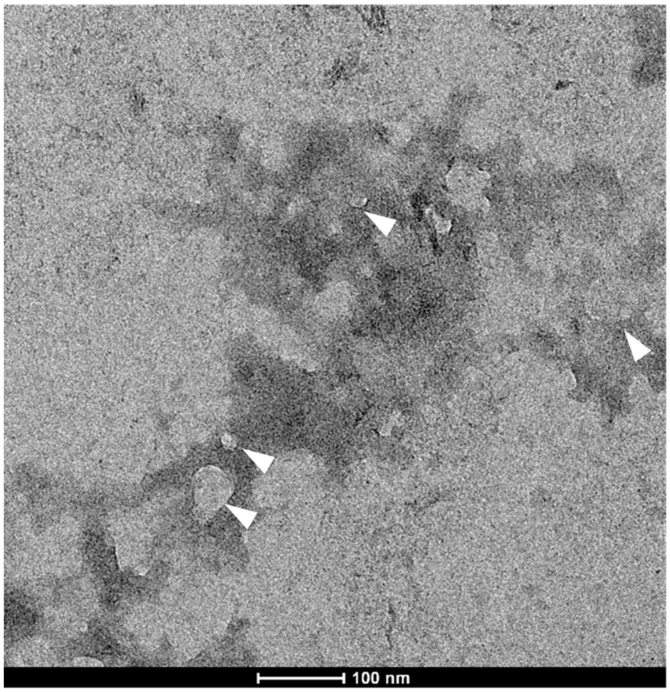

Figure S2. Low magnification TEM images of BEV clusters using a negative staining technique. White arrows indicate BEVs. Scale bar = 100 nm.

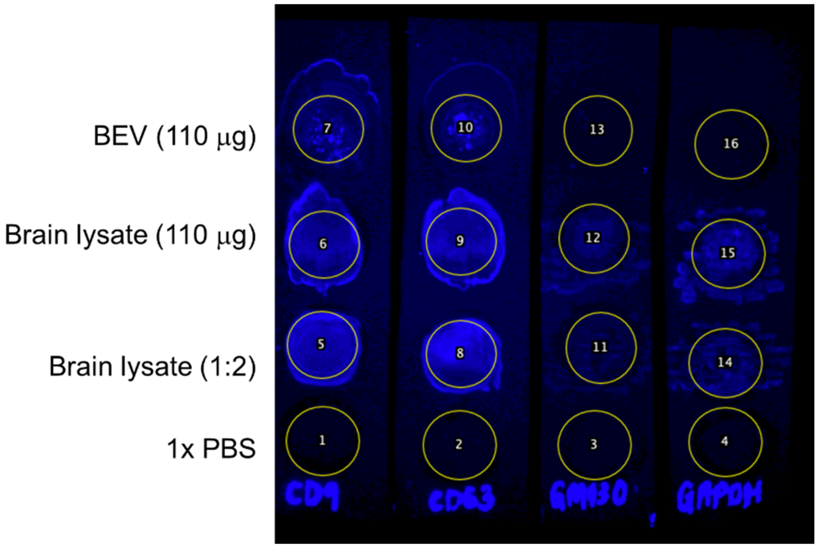

Figure S3. Quantification of dot immunoblots using ImageJ. The integral of signal density was quantified within regions of interest delineated with circles of the same area for each sample.

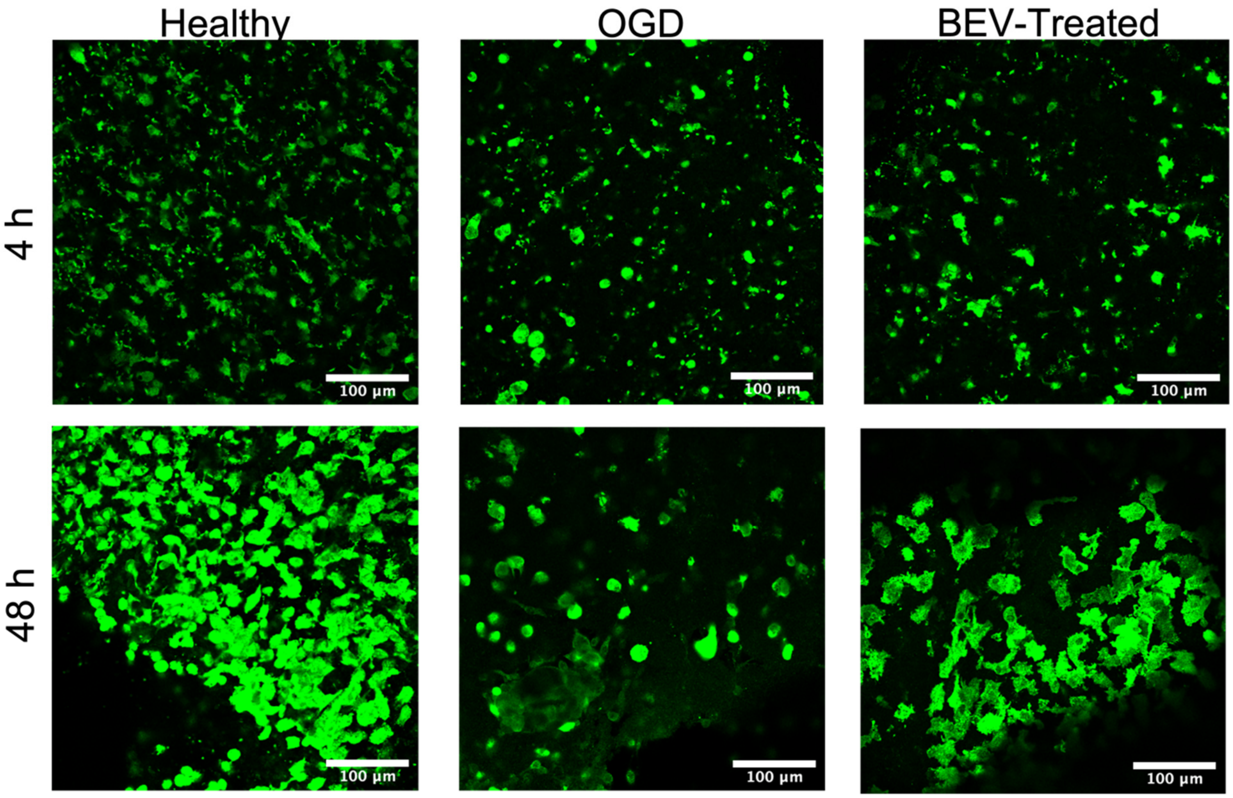

Figure S4. Representative confocal imaging examples of Iba-1-stained microglia at 40× magnification from the cortex of ex vivo brain slices for healthy, OGD control, and 25 μg BEV-treated slices at an exposure time of 4 and 48 h. Scale bar = 100 μm.

Treatment	Exposure	Shape Mode 1	Shape Mode 2	Shape Mode 3	Shape Mode 4	Shape Mode 5
non-treated control	0 hr	16.3	15	21.5	29.6	17.6
	4 hr	18.5	23.3	15.1	25.3	17.8
	24 hr	14.9	24.3	26	22.1	12.7
	48 hr	15.6	15.6	37.4	19	12.3
ogd control	0 hr	17.7	23.8	20.4	21.4	16.7
	4 hr	17.9	13.5	20.8	31.4	16.4
	24 hr	17.6	19.9	34.1	15.3	13.1
	48 hr	15.2	23.2	28	17.6	16
bev treatment	4 hr	12.3	20.3	29.5	21.2	16.7
	24 hr	12.2	21.8	36.5	17.3	12.2
	48 hr	22.5	17	21.7	21.7	17

Key: Percent Shape Mode	12	17	21	27	38

Figure S5. Global heatmaps of percent SM by treatment and then exposure time.

Table S1. Characterization data from BEV extractions used in dose- and time-efficacy studies. Data include size distribution, concentration, and purity. Size and particle number were measured by NTA, and purity was measured by BCA and NTA.

EV extract #	Mean Diameter (nm)	Mode Diameter (nm)	Particle #	Purity (Particle/mL)
1	279.3	227.5	2.73×10^{11}	5.76×10^{8}
2	155.8	132.3	2.82×10^{11}	4.35×10^{8}
3	197.3	144.2	3.63×10^{11}	9.65×10^{8}
4	208.4	138	2.52×10^{11}	4.00×10^{8}
5	197.8	139.2	2.14×10^{11}	5.46×10^{8}
6	216.6	129.5	6.17×10^{11}	1.43×10^{9}
7	210.2	140	2.77×10^{11}	4.09×10^{8}
8	189.3	145	4.42×10^{11}	4.11×10^{8}
9	281.7	180	6.42×10^{11}	7.14×10^{7}
10	146.1	202.5	3.96×10^{11}	6.91×10^{8}
